# Repetitive Transcranial Magnetic Stimulation: A Call for Better Data

**DOI:** 10.3389/fncir.2016.00057

**Published:** 2016-08-03

**Authors:** Marcus T. Wilson, Lynley St George

**Affiliations:** School of Engineering, University of WaikatoHamilton, New Zealand

**Keywords:** repetitive transcranial magnetic stimulation (rTMS), principal components analysis (PCA), motor evoked potential (MEP), synaptic plasticity (LTP/LTD), correlation analysis

## Introduction

Repetitive Transcranial Magnetic Stimulation (rTMS) is increasingly used to treat stroke, Parkinson's disease and depression (Fregni et al., 2005; Loo and Mitchell, 2005; Hallet, 2007; O'Reardon et al., 2007; Ridding and Rothwell, 2007). rTMS uses bursts of magnetic pulses to change the excitability and connection strengths of cortical neurons. However, the evidence to inform clinical application is highly inconsistent (Thut and Pascual-Leone, 2010; Hamada et al., 2013) and substantially based on trial and error. Systematic theory is lacking. Typically, in rTMS research, measurements of motor-evoked potential (MEP) are made, often in terms of the strength of the MEP and the length of the cortical silent period that follows. However, the MEP is probably a poor and certainly an indirect measure of changes in the brain (Nicolo et al., 2015), clouding our understanding of rTMS mechanisms. In practice, therefore, particular amplitudes and timing of pulses in an rTMS sequence are selected because they show promise in small subsets of people. However, even basics such as the sign of any change in the outcome measure (e.g., does the MEP increase or decrease?) is debated. Many results show a wide spread in responses. It has become common to talk about “responders” and “non-responders” although evidence for a binary distinction in these two groups is lacking—in reality there is usually a continuum of response often including potentiation in some and depression in others (Nettekoven et al., 2015). Moreover, Héroux et al. (2015) provide evidence that the irreproducibility of results may be due to small sample sizes, unscientific screening of subjects and data, and selective reporting of results.

In rTMS a regular train of pulses is applied. There is considerable variation in possible stimulation sequences, leading to many parameters that could potentially affect the results. Pulses are applied at a particular amplitude (normally recorded as percent motor threshold, (%RMT), or sometimes percent machine output, themselves imprecise measures), at a given rate (pulses per second, or hertz, Hz), until a particular number of pulses have been applied. There are three numerical parameters here. Additionally, one can consider variation in coil shape, orientation and place of application. Different waveforms for the magnetic pulse are also available. Thus, for an ostensibly straightforward pulse sequence such as non-bursting rTMS, the parameter space is considerable. Fitzgerald et al. (2006) carried out a comprehensive review of rTMS effects. They concluded that low frequency (0.9–5 Hz) stimulation generally results in a decreased MEP, while high frequency stimulation (5–20 Hz) results in an increased MEP. Very low frequency stimulation gave no effect. The “<5 Hz, depression; >5 Hz, potentiation” mantra is now well established in rTMS literature.

More recently, bursting protocols have become popular. The quadripulse protocol (Tsutsumi et al., 2014) is one example—four pulses are applied in quick succession, with this pattern repeated at regular time intervals. Theta-burst stimulation (TBS) is another; bursts of pulses are applied at theta-band (4–8 Hz) frequency. A continuous theta-burst stimulation (cTBS) protocol adds two more parameters, the number of pulses in a burst and the burst frequency. Intermittent theta-burst stimulation (iTBS) protocols require a further two parameters. Here, the bursting protocol is applied for a given time (denoted ON time, often 2 s), then removed for a given time (denoted OFF time, often 8 s) before being active for another 2 s period, etc. Thus, the parameter space for describing intermittent bursting pulse sequences becomes vast. Experiments performed to date do not come close to spanning it.

## Descriptions of previous results

Recently, there has been some focus on developing good numerical models of the effects of rTMS (Robinson, 2011; Fung et al., 2013; Wilson et al., 2014). That has been our primary motivation for revisiting previous rTMS experiments (particularly cTBS and iTBS) with a view to identifying overall trends. Which parameters are the most important? What, on average, are their effects? Various forms of statistical regression, such as Principal Component Analysis (PCA) should potentially be of benefit here. However, it quickly became clear that for bursting sequences (e.g., cTBS and iTBS) there is insufficient variation in data for such an analysis to be meaningful. Few protocols have been evaluated in the large parameter space. Recently, for example, the cTBS and iTBS protocols of Huang et al. (2005) have come to the fore and are being used to the substantial exclusion of other possibilities.

In the case of repetitive, but not bursting protocols, there is more variation, particularly in earlier studies. This has allowed us to tackle an analysis with regression and PCA. We have identified 92 publications looking at rTMS protocols (from 1994 to 2006) containing results of 164 different experiments. However, in many of these cases data were not systematically reported and key information was missing. From these publications we produced a subset of 35 publications containing data from 79 different experiments in which we could unambiguously identify the frequency of the rTMS, the number of pulses applied in the protocol, the intensity of the applied pulse (in terms of %RMT) and the overall effect on the MEP. These experiments covered 1064 different subjects (although some of these subjects are likely to be the same person). The number of subjects in these experiments ranged from four to 45, with a median of ten. Nineteen publications reported on “one-off” experiments with a single protocol, as opposed to multiple protocols used on the same set of subjects. Various clinical populations were covered, including Parkinson's disease, epilepsy, major depression and focal hand dystonia, in addition to healthy populations. We have not looked at the effect of population on results.

A few publications recorded the effect on the MEP in a quantitative manner; most did not. Rather, it has been usually recorded qualitatively as “increase,” “decrease,” “no change,” or, unhelpfully, “variable.” Again, there is a problem of interpretation here. What is it about the MEP that “increases”? Is it the amplitude of the MEP, the time-scale over which it occurs, or the integrated area of the MEP? Many publications are vague on this point. We have taken a pragmatic approach and simply left the effect on the MEP, for the purposes of analysis, as being “increase,” “decrease” or “no change.” We assigned each of these results the numeric values of 1, 0, and −1, respectively. Those protocols which recorded “variable” results, were assigned 0, although the results of analyses do not change much when these experiments are excluded.

We thus constructed a four-dimensional dataset recording frequency of rTMS (*f*), number of pulses applied (*N*), amplitude of each pulse (*A*), and (tri-valued) effect on the MEP, *M*. We then carried out two analyses. First, we used linear regression to find the overall effect of *f*, *N*, and *A* on *M*. Secondly, we used PCA to determine which variable combinations were most important for influencing *M* and their overall effect. To eliminate some bias, we weighted each experiment by the number of participants.

**Figure 1:**
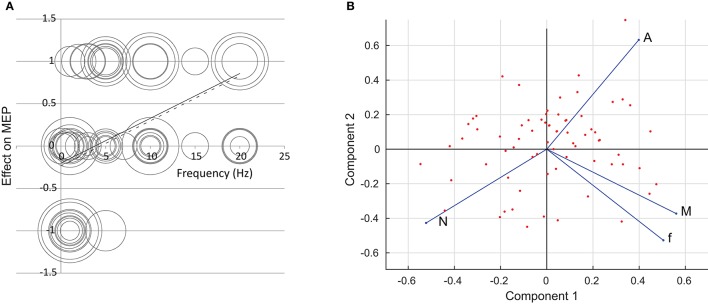
**(A)** The effect of repetition frequency *f* on the size of the MEP. The circles denote different experiments; the area of the circles is proportional to the number of participants in each experiment. The solid line is a result of linear regression; the dashed line is a result of linear regression where “one-off” experiments have been excluded. **(B)** A two-dimensional rendering of the rTMS data set using Principal Component Analysis. The first and second components form the *x*- and *y*-axes respectively. The points mark the individual experiments. The *f*, *N*, *A*, and *M* axes are shown in terms of the first two principal components. Data are taken from: Pascual-Leone et al., 1994; Jennum et al., 1995; Wassermann et al., 1996; Chen et al., 1997a,b; Berardelli et al., 1998, 1999; Siebner et al., 1999a,b; Maeda et al., 2000a,b; Muellbacher et al., 2000; Rollnik et al., 2000; Romeo et al., 2000; Siebner et al., 2000; Wu et al., 2000; Fierro et al., 2001; Lorenzano et al., 2002; Romero et al., 2002; Sommer et al., 2002; Cincotta et al., 2003; Gorsler et al., 2003; Grunhaus et al., 2003; Modugno et al., 2003; Schambra et al., 2003; Fitzgerald et al., 2004; Peinemann et al., 2004; Stinear and Byblow, 2004; Brighina et al., 2005; Fitzgerald et al., 2005; Murase et al., 2005; Quartarone et al., 2005a,b; Daskalakis et al., 2006; Inghilleri et al., 2006.

The regression analysis showed that it was only the pulse frequency *f* that had any overall effect on the size of the MEP. For frequency, the analysis is shown in Figure 1A. Here, the result is plotted against the applied frequency. The area of the circles is proportional to the number of subjects in each experiment. The solid line shows the results of linear regression (with each experiment weighted by the number of participants); the dashed line shows regression where the more questionable “one-off” experiments have been excluded. We acknowledge that linear regression is dubious since we have made the results tri-valued only and there is no *a priori* reason for believing a linear relationship should apply.

Results of PCA are summarized in Figure 1B. From a four-dimensional data-set we obtain four principal components. The components, in order, explain 42, 31, 16, and 11% of the variation in the data. We plot the data points in terms of the first two principal components, and show the direction of the *f*, *N*, *A* and *M* axes on the figure.

It is clear that frequency is the major driver of the change in the MEP. Figure 1A shows the effect is for low frequencies to reduce the MEP, and high frequencies to increase it, in line with the much-assumed *f* < 5 Hz, depression; *f* > 5 Hz, potentiation. However, the relationship is weak, with results widespread. This is supported by Figure 1B, which indicates that the *M* axis lies in a similar direction to the *f* axis, meaning that an increase in *f* leads in general to an increase in the end result *M*. Results show little link between *N* and *A* and the end result. On the PCA plot of Figure 1B, the *N* and *A* axes lie roughly perpendicular to the end result. Linear regression (not shown) gives no apparent trend.

## A request for more systematic data

We do not wish to draw too much from the above results, other than to say that *broadly speaking* they support the established rTMS dogma around the effect of repetition rate on MEP. That in itself is unsurprising given that Fitzgerald et al. (2006) comprehensively tabulated a large number of experiments, and there is considerable overlap between our datasets. Too often, however, the mantra “<5 Hz, depression; >5 Hz, potentiation” is stated without acknowledging the extent of the variation in results. Although Héroux et al. (2015) have exposed some questionable research practices, excluding the “one-off” data makes little difference to this conclusion.

The main focus of our comments is the difficulty we experienced in performing such analyses, notably our inability to analyze the cTBS and iTBS literature in a quantitative manner.

First, we note that the typical output measure of rTMS, the MEP, is poorly defined. It is clear that different authors mean different things by this. It is not just a case that electrical activity of different muscles are measured; rather that there is no quantitative or even consistent qualitative definition of the change in the MEP. We are left mostly with vague terminology “increase,” “decrease,” “no change,” or even “fluctuates.” If we are to start understanding the results, drivers and mechanisms of rTMS, we need to start by robustly defining outcome measures. A minority of publications have focused on the length of the cortical silent period (CSP, the quiet period of the electromyogram following a MEP) rather than the “size” of the MEP. This is more easily quantifiable and is a more direct measure of cortical effects (Ziemann et al., 2015), therefore is possibly a better place to start. Unlike the MEP, which depends on a network of cortical and non-cortical excitatory and inhibitory processes, the CSP originates in the cortex and is mediated by activation of GABA_A_ and GABA_B_ (Ziemann et al., 2015).

If one persists with measuring the MEP, one needs also to relate changes in the MEP to changes in the brain. The response to TMS depends not only on cortical excitability but also to excitability at a spinal level and the properties of corticospinal-motoneurone connections. Without suitable models of the process, it is difficult to relate changes in MEP to changes in the cortex, and start to untangle the effects of plasticity, neural excitability and gene expression that all could affect the results of a TMS (Pell et al., 2011). One is therefore left with costly and difficult animal experiments (Vahabzadeh-Hagh et al., 2012).

Next, we must become more rigorous in the planning, execution, recording and publication of our experiments. Too often, key details have been left out. This has particularly been evident in some of the earlier experiments. In compiling our data set, we had to make assumptions about particular experiments because data were not unambiguously given. Indeed, our data set for producing the Figure used only around half of the literature we looked at, because we could not reliably identify the data we required. Moreover, our analysis relies on published data; we cannot analyze unpublished results.

Finally, we note that in the last 10 years human experiments have focused on a very limited range of pulse sequences, such as the cTBS and iTBS of Huang et al. (2005). Most of the high dimensioned parameter space that bursting rTMS sequences provide has been largely ignored. A much larger range of protocols is needed in order to investigate effects systematically. It is true that the focus on these protocols is a result of their promise in clinical applications, but in terms of science, we are left little the wiser about what rTMS is actually doing.

## Author contributions

MW conceived the research, processed some of the data and wrote the majority of the article. LS identified the data set, carried out most of the data processing, identified the major issues and wrote the minority of the article.

### Conflict of interest statement

The authors declare that the research was conducted in the absence of any commercial or financial relationships that could be construed as a potential conflict of interest.
